# Comparative Diagnostic Sensitivity of Endoscopic Ultrasound‐guided Tissue Acquisition for Hepatocellular Carcinoma Versus Intrahepatic Cholangiocarcinoma

**DOI:** 10.1002/deo2.70423

**Published:** 2026-08-31

**Authors:** Yuichi Takano, Naoki Tamai, Jun Noda, Tetsushi Azami, Fumitaka Niiya, Ryo Uyama, Naotaka Maruoka, Tatsuya Yamagami, Akihiro Nakamura, Takafumi Ogawa, Takahiro Umemoto, Masatsugu Nagahama

**Affiliations:** ^1^ Department of Internal Medicine Division of Gastroenterology Showa Medical University Fujigaoka Hospital Yokohama Japan; ^2^ Department of Diagnostic Pathology Showa Medical University Fujigaoka Hospital Yokohama Japan; ^3^ Department of General and Gastroenterological Surgery Showa Medical University Fujigaoka Hospital Yokohama Japan

**Keywords:** endo‐hepatology, endoscopic ultrasound, hepatocellular carcinoma, intrahepatic cholangiocarcinoma, tissue acquisition

## Abstract

**Background and Aims:**

Accurate histological differentiation between hepatocellular carcinoma (HCC) and intrahepatic cholangiocarcinoma (ICC) is essential for selecting appropriate treatment. Endoscopic ultrasound‐guided tissue acquisition (EUS‐TA) is increasingly used for focal liver lesions; however, comparative data on its diagnostic sensitivity for HCC and ICC remain limited. This study compared the diagnostic sensitivity of EUS‐TA for these malignancies.

**Methods:**

This single‐center retrospective study included patients who underwent EUS‐TA for focal liver lesions and were ultimately diagnosed with HCC or ICC between 2016 and 2024. Patients with extrahepatic cholangiocarcinoma were excluded. The primary outcome was overall diagnostic sensitivity. Secondary outcomes were cytological sensitivity and procedure‐related adverse events.

**Results:**

Forty‐five patients were included (HCC, *n* = 18; ICC, *n* = 27). Median lesion size was 41 mm. Baseline characteristics were similar, except that fine‐needle biopsy needles were used less frequently in HCC than in ICC (16.6% vs. 48.1%, *p* = 0.032). Overall diagnostic sensitivity was high in both groups but lower in HCC than in ICC (83.3% [95% confidence interval {CI}, 58.6–96.4%] vs. 100% [95% CI, 87.2%–100%], *p* = 0.029). The difference was more pronounced for cytological sensitivity (22.2% [95% CI, 6.4%–47.6%] vs. 77.8% [95% CI, 57.7%–91.4%], *p* = 0.0007). No procedure‐related adverse events occurred.

**Conclusions:**

EUS‐TA demonstrated high diagnostic sensitivity for both HCC and ICC without procedure‐related adverse events. However, diagnostic sensitivity, particularly cytological sensitivity, was lower in HCC, consistent with the known pathological characteristics of HCC and the established role of histological evaluation in its diagnosis.

**Trial Registration**: N/A.

## 1 Introduction

Hepatocellular carcinoma (HCC) and intrahepatic cholangiocarcinoma (ICC) are the most prevalent primary liver malignancies, each with distinct therapeutic strategies and prognostic outcomes [1]. Although contrast‐enhanced computed tomography (CT) and magnetic resonance imaging (MRI), in combination with tumor marker assessment, often guide diagnosis, atypical imaging findings may necessitate histological confirmation, including immunohistochemical (IHC) evaluation [2].

Percutaneous liver biopsy has long been considered the gold standard for histological diagnosis [3]; however, its limitations—including pain, bleeding risk, and difficulty accessing certain hepatic segments—have prompted interest in alternative approaches. Recently, the concept of Endo‐Hepatology, advocated by Chang et al. [4], has gained recognition, with endoscopic ultrasound‐guided tissue acquisition (EUS‐TA) emerging as a promising diagnostic modality [5, 6]. A recent meta‐analysis reported a diagnostic yield of 92.4% for EUS‐TA in focal liver lesions [7]. However, most available studies have evaluated heterogeneous cohorts, often including a substantial proportion of metastatic liver lesions, without stratifying diagnostic performance according to tumor subtype. Direct head‐to‐head comparisons of EUS‐TA between HCC and ICC remain scarce.

Therefore, the present study aimed to compare the diagnostic sensitivity and safety of EUS‐TA for HCC and ICC.

## 2 Methods

This single‐center, retrospective, observational study was approved by the ethics committee of Showa Medical University (Approval number: 2026‐0192) and conducted in accordance with the Declaration of Helsinki.

This study included patients who underwent EUS‐TA for focal liver lesions between 2016 and 2024 and ultimately received a final diagnosis of HCC or ICC. Patients with extrahepatic cholangiocarcinoma were excluded.

Clinical characteristics and procedural details of EUS‐TA were retrospectively reviewed using medical records. The primary outcome was overall diagnostic sensitivity of EUS‐TA. Secondary outcomes included cytological sensitivity and procedure‐related adverse events.

Because all included patients were ultimately diagnosed with malignancy (HCC or ICC), specificity and overall diagnostic accuracy were not calculated, and diagnostic performance was assessed based on sensitivity.

### 2.1 EUS‐TA Procedure

During EUS‐TA, conscious sedation and analgesia were administered using pethidine hydrochloride (35 mg) or pentazocine (7.5–15 mg) in combination with midazolam (1.0–4.5 mg), at the discretion of the operator. A convex‐array echoendoscope (GF‐UCT260; Olympus Medical Systems, Tokyo, Japan) in combination with an ultrasound processor (UE‐ME1 or UE‐ME2; Olympus Medical Systems) was used.

Needle gauge (19–25 gauge) and needle type were selected at the operator's discretion. The needles used included Expect SlimLine (Boston Scientific Japan, Tokyo, Japan) for fine‐needle aspiration (FNA), and Acquire (Boston Scientific Japan) or SonoTip TopGain (Medico's Hirata, Tokyo, Japan) for fine‐needle biopsy (FNB). Typically, 10–20 to‐and‐fro movements were performed with 10–20 mL negative pressure suction. When significant blood contamination was observed, the slow‐pull technique was used instead. Rapid on‐site cytological evaluation was not performed.

In patients receiving antithrombotic agents, EUS‐TA was performed in accordance with the Japanese Gastrointestinal Endoscopy Society guidelines [8]. Contrast‐enhanced EUS was not routinely performed. All procedures were performed by experienced endosonographers.

### 2.2 Our Institutional Strategy for Focal Liver Lesions

At our institution, focal liver lesions with characteristic imaging findings and a definitive diagnosis generally do not undergo biopsy. Tissue acquisition is considered for lesions with atypical imaging features or when histological confirmation is required before treatment initiation, particularly in patients with a history of extrahepatic malignancy in whom metastatic liver disease is a diagnostic consideration. In cases where the indication for biopsy is uncertain, the need for tissue acquisition is determined through multidisciplinary discussion involving gastroenterologists, surgeons, oncologists, and radiologists.

EUS‐TA was considered the first‐line tissue acquisition approach for lesions located in the left hepatic lobe and caudate lobe. For right hepatic lobe lesions, EUS‐TA was performed when preprocedural CT demonstrated that EUS‐guided access was feasible.

EUS‐TA was also selected for lesions considered technically challenging for percutaneous liver biopsy, such as those located immediately beneath the diaphragm, or in patients with severe obesity.

### 2.3 Pathological Examination

Tissue specimens obtained by EUS‐TA were fixed in formalin and processed for histological evaluation using hematoxylin and eosin staining. IHC analysis was performed when necessary. The residual liquid specimen was submitted for cytological evaluation using Papanicolaou staining. This specimen‐processing approach is not unique to our institution and has been described in previous studies and pathological reviews of EUS‐TA [9, 10].

## 3 Definitions

### 3.1 Pathological Diagnosis

All pathological evaluations were performed by an experienced pathologist. Cytological and histological specimens were considered adequate if they allowed definitive pathological evaluation and inadequate if they did not. The reasons for inadequate specimens (e.g., insufficient material or tissue degeneration) were recorded.

For cytological evaluation, cases categorized as malignant or suspicious for malignancy were considered positive. For histological evaluation, cases diagnosed as HCC or ICC, as well as those considered suspicious for HCC or ICC, were defined as positive.

### 3.2 Final Diagnosis

Final diagnosis was established based on histopathological findings or clinicoradiological follow‐up. Cases were classified as HCC or ICC when histological diagnosis confirmed the tumor type and the subsequent clinical course was consistent with the diagnosis. Cases with an initial pathological diagnosis negative for malignancy were still classified as HCC or ICC if subsequent clinical follow‐up, including imaging progression, surgical pathology, or tumor marker elevation, confirmed the presence of malignancy.

### 3.3 Adverse Events

Procedure‐related adverse events were defined according to the American Society for Gastrointestinal Endoscopy (ASGE) lexicon [11].

### 3.4 Statistical Analysis

Continuous variables are expressed as medians and were compared using the Mann–Whitney U test. Categorical variables were compared using Fisher's exact test. Diagnostic sensitivity estimates were calculated with exact 95% confidence intervals using the Clopper–Pearson method. A two‐sided *p*‐value < 0.05 was considered statistically significant.

## 4 Results

During the study period, EUS‐TA initially diagnosed 15 patients with HCC and 27 with ICC. Among cases initially deemed negative for malignancy by EUS‐TA, three were ultimately diagnosed with HCC. In two of these cases, tumor progression led to surgical resection, and pathological examination confirmed well‐differentiated HCC. The remaining case was clinically diagnosed as HCC based on tumor progression and elevated alpha‐fetoprotein (AFP) levels. No initially negative cases were subsequently diagnosed with ICC. Consequently, 18 patients with HCC and 27 with ICC were included in the final analysis.

HCC is generally diagnosed based on characteristic imaging findings and serum tumor markers without the need for histological confirmation. Therefore, the HCC cases included in the present study represent a selected subgroup requiring pathological diagnosis. During the study period, a total of 216 patients were diagnosed with HCC at our institution. Histological confirmation was obtained in 21 patients (9.7%) (EUS‐TA, *n* = 18; percutaneous biopsy, *n* = 3). The present study included the 18 patients who underwent EUS‐TA.

Typical radiological findings, defined as arterial phase hyperenhancement with portal venous or delayed‐phase washout on dynamic CT, were observed in only seven of the 18 patients (38.9%), indicating that most patients had atypical imaging features. In addition, the median serum AFP and protein induced by vitamin K absence‐II (PIVKA‐II) levels were 4.2 ng/mL (range, 1.7–61,840) and 109 mAU/mL (range, 22–51,430), respectively. AFP levels were within the normal range in 14 patients (77.8%), while PIVKA‐II levels were within the normal range in 8 patients (44.4%), further highlighting the diagnostic difficulty of this cohort. The indications for histological diagnosis included atypical imaging findings (61.1% [11/18]), differentiation from metastatic liver tumors (38.9% [7/18]), and the need for pathological confirmation before systemic chemotherapy (27.8% [5/18]), with some patients having multiple indications.

Baseline characteristics are summarized in Table 1. There were no significant differences between the HCC and ICC groups in median age (74.5 vs. 73 years, *p* = 0.214), sex distribution (female, 11.1% vs. 33.3%, *p* = 0.210), or median tumor size (42.5 vs. 43 mm, *p* = 0.843). The left hepatic lobe was the most common tumor location in both groups. The proportions of right lobe lesions (33.3% vs. 22.2%, *p* = 0.414) and caudate lobe lesions (5.6% vs. 11.1%, *p* = 0.525) were comparable between groups. Non‐viral/non‐alcohol‐related liver disease was the most common underlying etiology in both groups.

**TABLE 1:** Comparison of clinical characteristics between hepatocellular carcinoma (HCC) and intrahepatic cholangiocarcinoma (ICC) patients.

	HCC group (*n* = 18)	ICC group (*n* = 27)	*p*‐value
Age, median (range), years	74.5 (57–90)	73 (30–85)	0.214
Female, no. (%)	2 (11.1)	9 (33.3)	0.21
Size, median (range), mm	42.5 (16–191)	43 (10–170)	0.843
Left lobe lesions, no. (%)	11 (61.1)	18 (66.7)	0.706
Right lobe lesions, no. (%)	6 (33.3)	6 (22.2)	0.414
Caudate lobe lesions, no. (%)	1 (5.6)	3 (11.1)	0.525
Etiology of liver disease			
HBV infection, *n* (%)	0	2 (7.4)	0.242
HCV infection, *n* (%)	2 (11.1)	0	0.179
Alcohol‐related, *n* (%)	6 (33.3)	5 (18.5)	0.263
Non‐viral/non‐alcoholic, *n* (%)	10 (55.5)	20 (74.1)	0.201
EUS‐TA procedure details			
Transduodenal puncture, no. (%)	5 (27.8)	7 (25.9)	0.891
19‐gauge needle, no. (%)	0	4 (14.8)	0.09
22‐gauge needle, no. (%)	12 (66.6)	14 (51.8)	0.329
25‐gauge needle, no. (%)	6 (33.3)	9 (33.3)	1
FNB needle, no. (%)	3 (16.6)	13 (48.1)	0.032
Needle passes, median (range)	2 (1–3)	2(1–2)	0.912

The proportion of transduodenal biopsies was similar between the HCC and ICC groups (27.8% vs. 25.9%, *p* = 0.891). FNB needles were used significantly more frequently in the ICC group than in the HCC group (48.1% vs. 16.6%, *p* = 0.032). A 22‐gauge needle was the most frequently used in both groups, with no significant difference in needle gauge distribution. The median number of needle passes was 2 in both the HCC and ICC groups (range, 1–3 vs. 1–2, respectively), with no significant difference between the groups (*p* = 0.912).

All cytological specimens were adequate for evaluation. One histological specimen in the HCC group was inadequate because of insufficient material; all remaining histological specimens were adequate.

**FIGURE 1:**
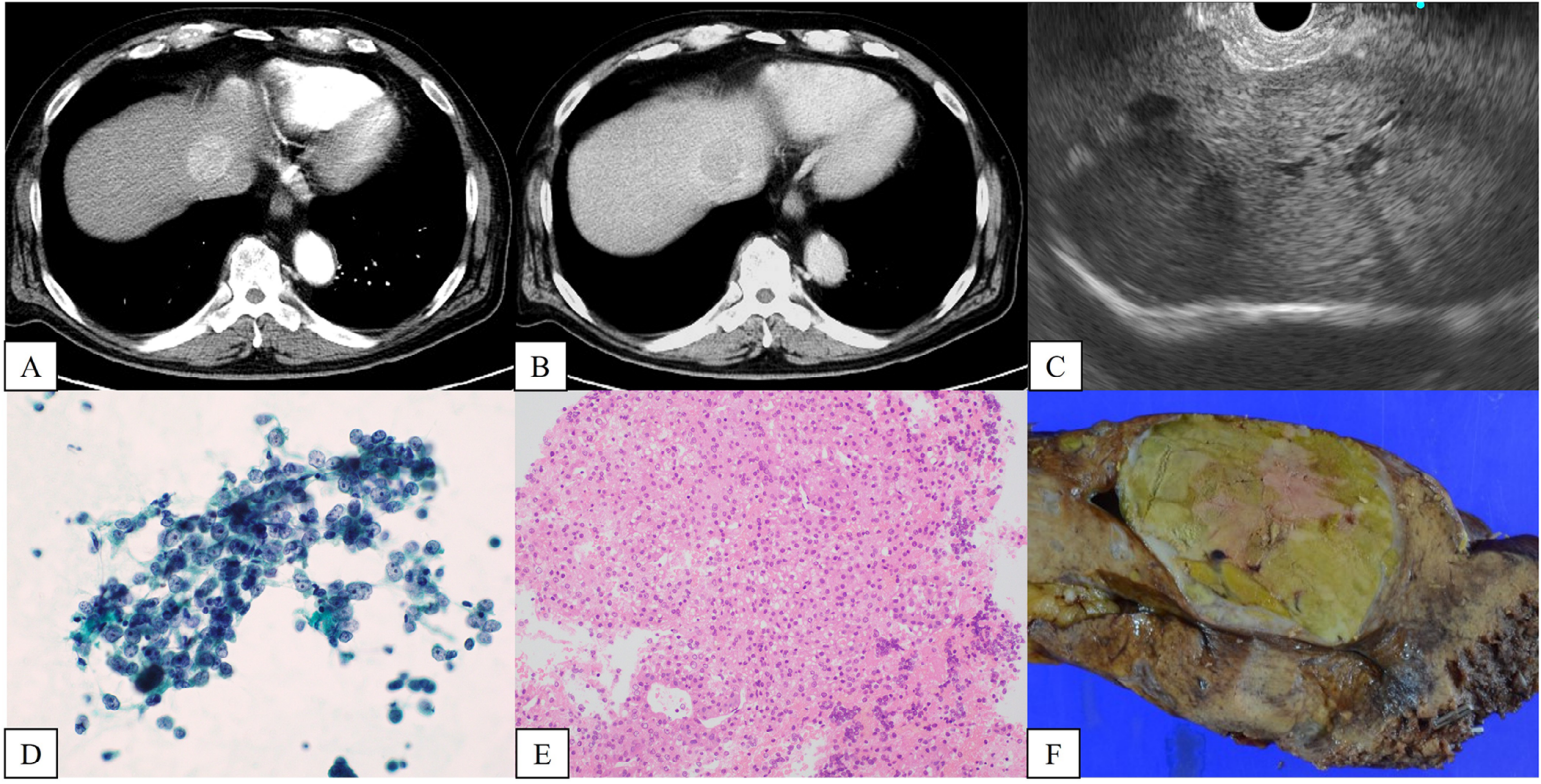
(A) Contrast‐enhanced computed tomography (CT) (arterial phase) shows an approximately 2‐cm enhancing tumor in segment 4 of the liver. (B) In the portal venous phase, the lesion shows washout, raising suspicion of hepatocellular carcinoma (HCC). However, because the patient has a history of renal cell carcinoma, differentiation from hepatic metastasis is required. (C) Endoscopic ultrasound‐guided tissue acquisition (EUS‐TA) is performed from the duodenal bulb using a 25‐gauge fine‐needle biopsy needle. (D) Cytological evaluation yields an intermediate result (Class III), and a definitive diagnosis of malignancy cannot be established. (E) Histological examination confirms HCC. (F) Following left portal vein embolization, left hepatectomy is performed. Gross examination shows a well‐demarcated yellow tumor, and histology confirms well‐differentiated HCC.

**FIGURE 2:**
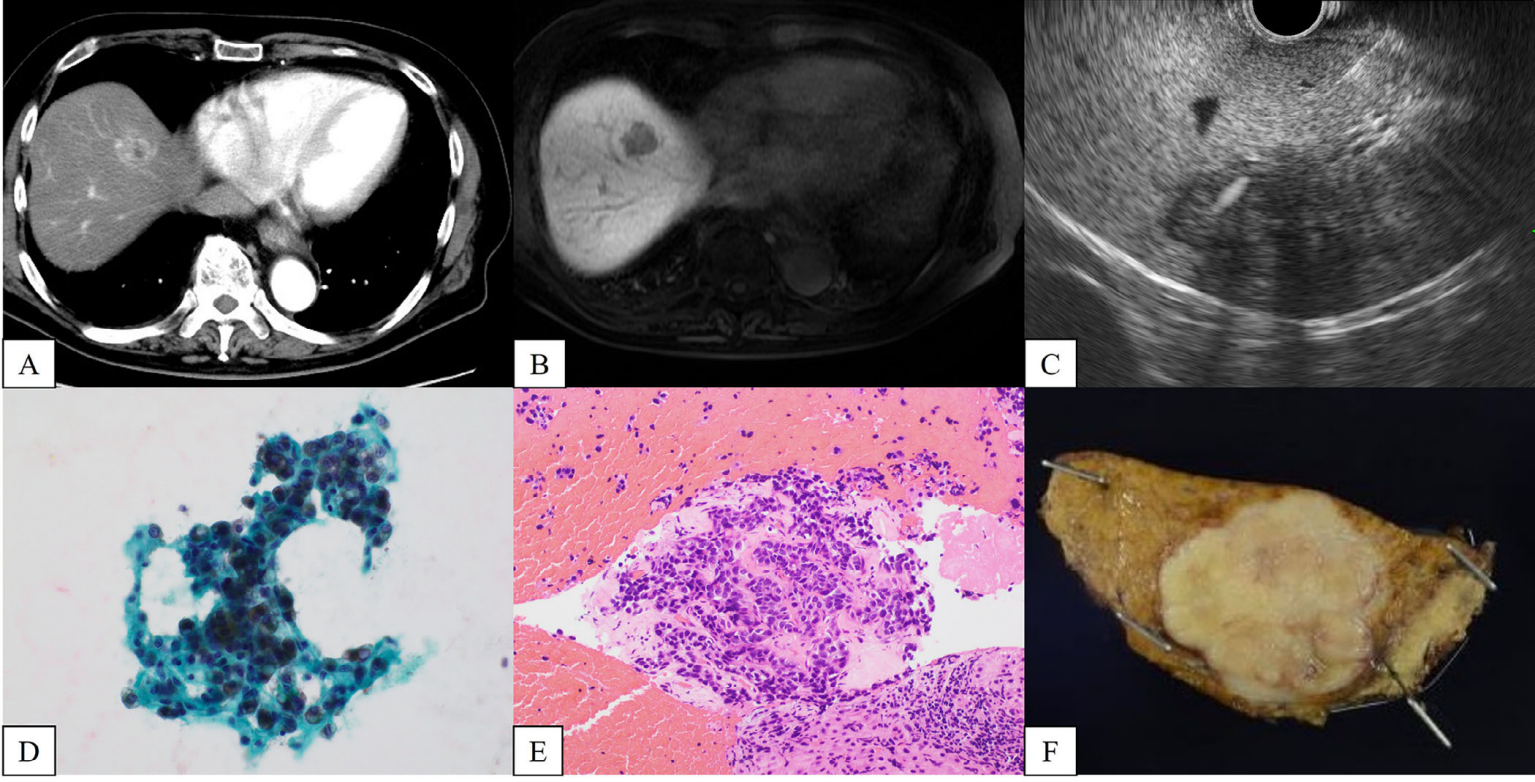
(A) Contrast‐enhanced computed tomography (CT) (arterial phase) shows a 15‐mm enhancing mass in segment 4 of the liver. The patient has a history of esophageal squamous cell carcinoma, raising the differential diagnosis of hepatic metastasis, hepatocellular carcinoma (HCC), and intrahepatic cholangiocarcinoma (ICC). (B) Gadoxetic acid‐enhanced magnetic resonance imaging (MRI) (hepatobiliary phase) shows a hypointense lesion in segment 4. (C) Endoscopic ultrasound from the duodenal bulb reveals an irregular hypoechoic mass. Endoscopic ultrasound‐guided tissue acquisition (EUS‐TA) is performed using a 22‐gauge fine‐needle biopsy needle. (D) Cytological evaluation confirms adenocarcinoma. (E) Histological examination reveals malignant cells consistent with cholangiocarcinoma, leading to the diagnosis of intrahepatic cholangiocarcinoma. (F) Gross examination of the resected specimen shows a whitish, irregularly marginated nodule, and histology confirms intrahepatic cholangiocarcinoma.

Overall diagnostic sensitivity was high in both groups, although it was modestly lower in the HCC group than in the ICC group (83.3% [95% confidence interval {CI}, 58.6–96.4%] vs. 100% [95% CI, 87.2%–100%], *p* = 0.029). The difference was particularly pronounced in cytological sensitivity, which was significantly lower in the HCC group than in the ICC group (22.2% [95% CI, 6.4–47.6%] vs. 77.8% [95% CI, 57.7–91.4%], *p* = 0.0007). Histological sensitivity was likewise lower in the HCC group (83.3% [95% CI, 58.6–96.4%] vs. 100% [95% CI, 87.2%–100%], *p* = 0.029). No procedure‐related adverse events, including bleeding, perforation, or infection, were observed in either group (Table 2; Figures 1 and 2).

**TABLE 2:** Comparison of endoscopic ultrasound‐guided tissue acquisition (EUS‐TA) outcomes between hepatocellular carcinoma (HCC) and intrahepatic cholangiocarcinoma (ICC) patients.

	HCC group (*n* = 18)	ICC group (*n* = 27)	*p*‐value
Cytological sensitivity, % (95% CI)	22.2 (6.4–47.6)	77.8 (57.7–91.4)	0.0007
Histological sensitivity, % (95% CI)	83.3 (58.6–96.4)	100 (87.2–100)	0.029
Overall diagnostic sensitivity, % (95% CI)	83.3 (58.6–96.4)	100 (87.2–100)	0.029
Procedure‐related adverse events, *n* (%)	0 (0)	0 (0)	>0.99

Among the 15 HCCs successfully diagnosed by EUS‐TA, six were classified as well‐differentiated and 9 as moderately to poorly differentiated HCC. Among the three false‐negative cases, two were ultimately diagnosed as well‐differentiated HCC after surgical resection, whereas the remaining patient did not undergo surgical resection; therefore, the histological grade could not be determined. Overall, the final histological differentiation was well‐differentiated in eight patients, moderately to poorly differentiated in nine patients, and undetermined in one patient.

Among the three false‐negative HCC cases, a 22‐gauge FNA needle was used in two cases, and a 25‐gauge FNB needle was used in one case. In an exploratory subgroup analysis according to needle type, the diagnostic sensitivity remained numerically lower for HCC than for ICC with both FNA needles (86.7% [13/15] vs. 100% [14/14]) and FNB needles (66.7% [2/3] vs. 100% [13/13]). In the HCC group, although the difference was not statistically significant, diagnostic sensitivity tended to be lower for right‐lobe lesions than for left‐lobe lesions (66.7% [4/6] vs. 91.7% [11/12]). In contrast, diagnostic sensitivity was identical between the 22‐ and 25‐gauge needle groups (83.3% [10/12] vs. 83.3% [5/6]). These findings should be interpreted with caution because of the limited sample size.

Among the false‐negative HCC cases, one specimen was considered inadequate because of insufficient material and was therefore nondiagnostic. In the remaining two cases, the specimens were judged to be adequate for histopathological evaluation, and only mild fragmentation was observed. However, no definite malignant hepatocellular components were identified, resulting in false‐negative diagnoses.

Comprehensive genomic profiling using FoundationOne CDx was performed in two patients with ICC. Both specimens were successfully analyzed.

## 5 Discussion

HCC and ICC are the two most common primary liver malignancies, but their treatment strategies and prognoses differ substantially, making accurate differential diagnosis clinically essential. Although typical imaging findings often allow noninvasive diagnosis, atypical presentations remain diagnostically challenging. In patients with a history of extrahepatic malignancy, differentiation from metastatic liver lesions is also required. In addition, the increasing proportion of HCC attributable to non‐viral etiologies has further complicated diagnosis [12, 13]. According to the American Association for the Study of Liver Diseases guidelines, biopsy is recommended for suspected HCC in patients without cirrhosis or at‐risk chronic hepatitis B infection, because noninvasive imaging criteria are less reliable in these populations [2]. In our cohort, 55.5% of patients with HCC had non‐viral/non‐alcohol‐related liver disease, which may have contributed to diagnostic difficulty.

In recent years, EUS‐TA has gained increasing attention as an alternative to percutaneous biopsy for focal liver lesions. EUS‐TA allows precise real‐time targeting with Doppler‐guided vascular avoidance and provides access to anatomically challenging lesions, including the caudate lobe [14, 15]. Because EUS‐TA does not require skin puncture and is generally performed under sedation, it may be associated with less procedural pain than percutaneous biopsy [6, 16]. Furthermore, EUS‐TA has been reported to be feasible even in patients with ascites, a condition generally considered a relative contraindication to percutaneous biopsy [17]. Reported diagnostic yields for EUS‐TA in focal liver lesions generally range from approximately 75% to 100%, with adverse event rates of up to 6% [18]. However, most previous studies have evaluated heterogeneous patient populations, often including a substantial proportion of metastatic liver lesions, without stratifying outcomes according to tumor subtype.

Few studies have specifically examined the role of EUS‐TA in HCC. Singh et al. reported that EUS‐TA achieved 94% diagnostic accuracy in a small cohort of focal liver lesions, including eight cases of HCC, and detected significantly more lesions than transabdominal ultrasonography, CT, and MRI [19]. Chen et al. reported 90% diagnostic accuracy for EUS‐guided sampling in patients with suspected left‐lobe HCC who were unsuitable for percutaneous biopsy [20]. In contrast, published evidence regarding cholangiocarcinoma has largely focused on biliary strictures rather than intrahepatic mass lesions. Mohamadnejad et al. reported an overall diagnostic sensitivity of 73% for EUS‐TA in cholangiocarcinoma, with significantly higher sensitivity in distal than proximal tumors [21]. Onda et al. reported that EUS‐TA achieved a sensitivity of 89% and a diagnostic accuracy of 87% in patients with suspected cholangiocarcinoma presenting as hilar or extrahepatic biliary strictures [22], while a meta‐analysis reported pooled sensitivity and specificity of 80% and 97%, respectively, for malignant biliary strictures [23]. Many of these studies included extrahepatic cholangiocarcinoma, in which EUS‐TA is often technically more challenging.

The principal strength of this study is its exclusive focus on primary liver malignancies, specifically HCC and ICC. In our cohort, overall diagnostic sensitivity was significantly lower in HCC than in ICC (83.3% vs. 100%, *p* = 0.029), with particularly poor cytological sensitivity in HCC (22.2%). These findings suggest that cytology alone may have limited diagnostic utility when HCC is suspected.

The lower diagnostic sensitivity observed in HCC may partly reflect underlying histopathological characteristics. ICC typically demonstrates marked cytological and architectural atypia, facilitating diagnosis on both cytology and histology. In contrast, well‐differentiated HCC often exhibits subtle cytological atypia and preserved trabecular architecture, making definitive diagnosis more difficult, particularly when specimen volume is limited or fragmented [1]. In our study, two of the three false‐negative HCC cases were ultimately confirmed as well‐differentiated HCC by surgical pathology, supporting this explanation.

Specimen fragmentation may negatively affect the diagnostic performance of EUS‐guided liver biopsy. In the present study, one of the three false‐negative HCC cases yielded inadequate material, whereas the remaining two specimens were considered adequate for histopathological evaluation despite mild fragmentation. Because no malignant hepatocellular components were identified in either specimen, the diagnostic failure was more likely attributable to limited lesional tissue than to specimen fragmentation. Furthermore, tissue yield may be limited when a 25‐gauge needle is used, potentially reducing the likelihood of obtaining representative lesional tissue.

Procedural factors may also have contributed. FNB needles were used significantly more frequently in ICC cases, and previous studies have suggested that FNB improves tissue architecture preservation and diagnostic performance in focal liver lesions [24]. The excellent diagnostic sensitivity observed in ICC may partly reflect study design. Because extrahepatic cholangiocarcinoma was excluded, all ICC lesions in this study were intrahepatic masses, which were likely more technically accessible than biliary strictures.

Although no bleeding‐related adverse events were observed in the present study, including in patients with hypervascular HCC, the sample size was limited, and the risk of hemorrhage associated with needle biopsy of hypervascular liver tumors cannot be completely excluded. Therefore, EUS‐TA should be reserved for carefully selected patients in whom histological confirmation is essential for determining the appropriate treatment strategy after careful consideration of the potential risks and benefits. Such patients may include those with atypical imaging findings, inconclusive or non‐elevated tumor marker levels, or cases in which differentiation from metastatic liver tumors or other malignancies is necessary.

This study has several limitations. First, it was a single‐center retrospective study with a relatively small sample size. Second, needle selection was not standardized and may have introduced procedural bias. Finally, because all included patients were ultimately diagnosed with malignancy, specificity and negative predictive value could not be assessed.

In conclusion, although EUS‐TA achieved high overall diagnostic performance, cytological evaluation alone was significantly less sensitive for HCC than for ICC. Histological evaluation significantly improved the diagnostic sensitivity for HCC. These findings were consistent with the known pathological characteristics of HCC, including the limitations of cytological evaluation alone and the established role of histological assessment in its diagnosis.

## Author Contributions

**Yuichi Takano**: conception and design of the report; acquisition, analysis, and interpretation of the data; drafting of the final manuscript. **Naoki Tamai**, **Jun Noda**, **Tetsushi Azami**, **Fumitaka Niiya**, **Ryo Uyama**, **Naotaka Maruoka**, **Tatsuya Yamagami**, **Akihiro Nakamura**, **Takafumi Ogawa**, **Takahiro Umemoto**, and **Masatsugu Nagahama**: analysis and interpretation of the data and drafting of the final manuscript. All the authors have read and approved the final manuscript.

## Funding

The authors have nothing to report.

## Ethics Statement

**Approval of the research protocol by an Institutional Reviewer Board**: This is a single‐center, retrospective, observational study was approved by the ethics committee of Showa Medical University (Approval number: 2026‐0192).

## Conflicts of Interest

The authors declare no conflicts of interest.

## Data Availability

The data that support the findings of this study are available from the corresponding author upon reasonable request.
